# A Gender Lens on User Quality Ratings From Young Teenagers Assessing the Sun Safe App: Comparing Responses From Co-researchers and Participants of Pilot Intervention Studies

**DOI:** 10.2196/35203

**Published:** 2022-08-18

**Authors:** Isabelle M Clare, Jacinta Francis, Nisali Gamage, Rebecca Nguyen, Shelley Gorman

**Affiliations:** 1 Telethon Kids Institute University of Western Australia Perth Australia

**Keywords:** app development, co-design, sun exposure, sun protection, teenager, uMARS, UV index, vitamin D, young adolescents, sunburn, adolescent, smartphone, gender, sun, protection, app, engagement, sunburn, risk, melanoma, decision-making

## Introduction

We developed the iOS smartphone app *Sun Safe* to support healthy sun practices in young teenagers (aged 12-13 years) [1]. The production involved co-design with young *co-researchers* (ie, aged 12-13 years) with a health message of using sun protection when the UV index is ≥3 [1]. Important features include real-time and location-specific weather data on the UV index and gamified educational content [1,2].

We were concerned that indifferent attitudes expressed by male co-researchers during the development of *Sun Safe* [3] would translate into gendered differences in user quality ratings. Furthermore, we wondered whether involvement in the co-design process could bias quality assessments. The results presented in this letter compare the responses of co-researchers [1] with those of participants of the pilot intervention studies [4].

## Methods

All methods underpinning the development of the app and pilot intervention studies are described elsewhere [1,4]. Data were collected from co-researchers (n=15, 9 female and 6 male co-researchers) involved in the co-design of *Sun Safe* across a 10-month period (2018-2019) via telephone interviews or 2-hour in-person workshops (3 were run) [1]. Data were collected from participants (n=24, 17 female and 7 male participants) of placebo-controlled pilot intervention studies, which tested *Sun Safe* for 6 weeks (2020) [4]. Co-researchers downloaded and used the beta version of *Sun Safe* (via *TestFlight*) for 20 minutes during the final workshop (June 18, 2019) [1]. Pilot study participants accessed the fully developed app (v1.0.1, 2020) for 6 weeks in 2020 [4]; they also identified their gender (male, female, other, prefer not to say), age, and postcode of residence during recruitment. User quality ratings data were collected using the User Version of the Mobile Application Rating Scale (uMARS) [4].

## Results

There were twice as many recruited female participants (n=26) as male participants (n=13). Co-researchers were older (mean 13.8, SD 0.4 years) than pilot study participants (mean 12.7, SD 0.4 years). Most co-researchers used the app for 5-10 minutes (8/15, 53%); most pilot study participants used it every day or on most days (13/24, 55%).

Female co-researchers responded to more questions than male co-researchers (Table 1). Within *subjective quality* and *perceived impact*, male pilot study participants rated the *Sun Safe* app higher for *overall star rating* and *help-seeking* behaviors (Table 1).

**Figure 1:**
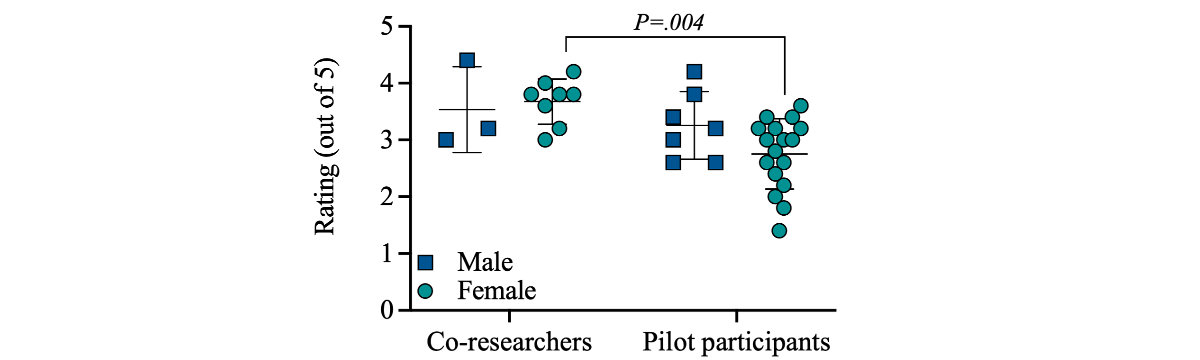
Female pilot study participants rated Sun Safe lower in the engagement area of assessment. Mean scores for questions asked across the engagement area of assessment are shown individually for each co-researcher (3 male and 8 female) and pilot study (7 male and 17 female) participant. Data are shown as mean (SD). Two-way ANOVA was used to compare differences (participant type x gender), with Tukey post hoc tests identifying a statistically significant difference in predicted means of 0.92 (95% CI 0.24-1.60; *P*=.004) between female co-researchers and female pilot study participants. The five questions were posed, and 5-point Likert scales within this area of assessment were as previously published.

Female pilot participants scored *Sun Safe* lower for *engagement* than female co-researchers (Figure 1).

**Table 1:** User quality ratings (User Version of the Mobile Application Rating Scale survey results) of the Sun Safe app for the subjective quality and perceived impact areas of assessment.

			Co-researchers			Pilot study participants		
			Male^a^	Female	*P* value	Male	Female	*P* value
Participants, n			6	9	N/A^b^	7	17	N/A
Questions completed^c,d^, n/N (%)			89/156 (57.1)	226/234 (96.6)	N/A	181/182 (99.5)	442/442 (100.0)	N/A
**Subjective quality^e,f^**								
	Recommended^g^, mean (SD)		3.3 (2.1)	3.6 (0.5)	.63	3.7 (1.1)	3.1 (1.1)	.26
	App use^h^, mean (SD)		3.0 (1.7)	4.1 (0.8)	.24	3.9 (0.7)	3.3 (1.2)	.37
	**Pay for app?^i^, n**							
		Yes	0	3	N/A	0	3	N/A
		No	3	6	N/A	7	14	N/A
	Overall star rating^j^, mean (SD)		4.2 (0.5)	3.6 (0.8)	.15	4.7 (0.7)	3.2 (0.9)	<.001
**Perceived impact^e,k^, mean (SD)**								
	Awareness^l^		3.7 (0.6)	3.7 (0.8)	>.99	4.0 (0.5)	3.4 (1.1)	.17
	Knowledge^m^		4.0 (0.0)	4.0 (0.7)	>.99	3.9 (0.7)	3.4 (1.2)	.54
	Attitudes^n^		3.0 (0.0)	3.4 (1.1)	.75	3.6 (0.8)	3.4 (0.9)	.79
	Intention to change^o^		3.3 (0.6)	3.9 (0.8)	.41	4.1 (0.9)	3.4 (1.1)	.17
	Help-seeking^p^		3.7 (1.2)	3.7 (0.7)	>.99	3.9 (1.1)	2.7 (1.0)	.04
	Behavior change^q^		4.0 (1.0)	3.7 (1.0)	.75	4.0 (0.8)	3.1 (1.1)	.09

^a^Two male participants did not complete any questions.

^b^N/A: not applicable.

^c^Total number of questions completed; 26 questions could be completed within the User Version of the Mobile Application Rating Scale (uMARS) survey by each participant.

^d^Percentage of questions completed of total possible (= total number completed by all participants / (n × 26) × 100), with statistical comparisons of the total number of uMARS survey questions completed (of 26), using Fisher Exact test, between male and female co-researchers (relative risk [RR] 0.60, 95% CI 0.50-0.70; *P*<.001) and pilot study participants (RR 0.99, 95% CI 0.97-1.00; *P*=.29).

^e^The *P* values are the results of Mann-Whitney tests comparing data by gender (except for *Pay for app?*).

^f^Across 4 questions, participants rated the subjective quality of the app, using 5-point scales (see below) or yes/no for *Pay for app?*

^g^Would you recommend this app to people who might benefit from it? (from 1, not at all, to 5, definitely).

^h^How many times do you think you would use this app in the next 12 months? (from 1, none, to 5, >50 times).

^i^Would you pay for this app? Yes is the number of participants answering *yes*; no is the number of participants answering *no*.

^j^What is your overall star rating of the app? (from * to *****; *One of the worst apps I’ve used* to *One of the best apps I’ve used*).

^k^Across 6 questions, participants rated the app based upon perceived capacity to modify awareness, knowledge, attitudes, intention to change, likelihood to seek help, and behaviors related to their sun health, using a 5-point scale of *strongly disagree* (1) to *strongly agree* (5).

^l^This app has increased my awareness of the importance of addressing sun health behaviors.

^m^This app has increased/changed my knowledge of sun health behaviors.

^n^This app has changed my attitudes toward improving my sun health behaviors.

^o^This app has increased my intentions/motivation to address my sun health behaviors.

^p^This app would encourage me to seek further help to address my sun health behaviors (if needed).

^q^Use of this app will change my sun health behaviors.

## Discussion

Overall, few differences in app quality ratings were observed by gender, suggesting that *Sun Safe* was equally acceptable for use by young men and women even though fewer male participants were recruited to develop and test *Sun Safe* [1]. Pilot study participants rated *Sun Safe* lower for *engagement*, highlighting the importance of an independent review. Limitations included the relatively small sample size, differences in review time, and ongoing challenges in defining the influences of biological sex and gender on health outcomes [5]. Additional consumer engagement will help determine how games and gamification could be further built into *Sun Safe*.
